# Evaluation of FeMnN alloy bioresorbable flow diverters in the rabbit elastase induced aneurysm model

**DOI:** 10.3389/fbioe.2025.1522696

**Published:** 2025-02-25

**Authors:** Alexander A. Oliver, Cem Bilgin, Jonathan Cortese, Esref A. Bayraktar, Daying Dai, Yong Hong Ding, Kent D. Carlson, Adam J. Griebel, Jeremy E. Schaffer, Mitchell L. Connon, Dan Dragomir-Daescu, Ramanathan Kadirvel, Roger J. Guillory, David F. Kallmes

**Affiliations:** ^1^ Biomedical Engineering and Physiology, Mayo Clinic Graduate School of Biomedical Sciences, Rochester, MN, United States; ^2^ Radiology, Mayo Clinic, Rochester, MN, United States; ^3^ Physiology and Biomedical Engineering, Mayo Clinic, Rochester, MN, United States; ^4^ Interventional Neuroradiology (NEURI Vascular Center), Bicetre University Hospital, Le Kremlin-Bicetre, France; ^5^ Fort Wayne Metals, Fort Wayne, IN, United States; ^6^ Biomedical Engineering, Medical College of Wisconsin, Milwaukee, WI, United States; ^7^ Neurologic Surgery, Mayo Clinic, Rochester, MN, United States

**Keywords:** flow diverter, bioresorbable, bioabsorbable, biodegradable, stent, aneurysm

## Abstract

**Introduction:**

Flow diverters are specialized stents used to treat intracranial aneurysms. Bioresorbable flow diverters (BRFDs) have been proposed as the next-generation of flow diverter technology. BRFDs aim to occlude and heal the aneurysm before safely dissolving into the body, mitigating complications associated with the permanent presence of conventional flow diverters. We previously prototyped BRFDs using an iron-manganese-nitrogen (FeMnN) alloy and demonstrated their flow diversion functionality, radial strength, bioresorbability, and MRI compatibility in benchtop tests. In the current work, we investigated their aneurysm occlusion efficacy *in vivo*.

**Methods:**

Elastase induced aneurysms were created in seven rabbits and BRFDs were deployed over the aneurysms for 3 months. Aneurysm occlusion efficacy and the biological response was assessed via angiography, gross dissection microscopy, and histology.

**Results:**

The BRFDs failed to occlude the aneurysms in 5/7 rabbits at the 3-month endpoint. The bioresorbable wires appeared to resorb too rapidly and fragment away from the aneurysm neck prior to becoming entirely encased in tissue and completely occluding the aneurysm. In 3/7 rabbits, some FeMnN wires remained over the aneurysm neck that were encased in tissue, partially covering the aneurysm neck. Histological analysis revealed that the wires, when present, were a suitable substrate over which tissue could develop. Therefore, we attribute the poor aneurysm occlusion efficacy to mechanical failure rather than an impaired biological healing response.

**Conclusion:**

The FeMnN BRFDs degraded too rapidly to effectively treat the rabbit elastase induced aneurysms. Future work will focus on developing BRFDs out of materials with a delayed resorption rate.

## 1 Introduction

An aneurysm is the excessive ballooning of an artery wall due to its weakening over time. This weakening makes aneurysms susceptible to rupture, which results in the loss of blood supply and infarction of downstream tissues. Intracranial aneurysms are estimated to be present in ∼5% of the US population (King, 1997). Approximately 30,000 aneurysms rupture annually with devastating consequences, as ∼50% of the patients die within 6 months and a high percentage of surviving patients are left with neurological impairments (King, 1997). Flow diverters (FDs) are a rapidly growing endovascular approach for the treatment of intracranial aneurysms. FDs are specialized stents composed of a high density of braided wires (Oliver et al., 2024). FDs are deployed in the parent artery over the neck of the aneurysm. They function by diverting the majority of blood flow away from the aneurysm sac, resulting in the stagnation of blood and the formation of a thrombus plug within the sac. In addition to diverting blood flow, the device also acts as a scaffold for tissue to grow over the aneurysm neck, ultimately occluding the aneurysm from blood flow (Kadirvel et al., 2014). The clinical use of FDs to treat aneurysms is growing due to their relatively high aneurysm occlusion rates (Brinjikji et al., 2013).

In patients, intracranial aneurysms treated with FDs typically take ∼6–12 months to occlude (Becske et al., 2017). However, all market approved FDs are composed of permanent materials (Dandapat et al., 2021) that will remain in the patient for the duration of their life, long after the device has served its intended purpose. Their long term presence is associated with several complications including device induced thromboembolism (Becske et al., 2017; Kallmes et al., 2015), stenosis (Guédon et al., 2016; Caroff et al., 2018; Flood et al., 2015), occlusion of adjacent branching arteries (Brinjikji et al., 2013), and metal induced imaging artifacts that may impede follow up computed tomography and magnetic resonance imaging (Halitcan et al., 2021; Bouillot et al., 2019). Bioresorbable flow diverters (BRFDs) have been proposed as the next-generation of FD technology (Oliver et al., 2022a; Jamshidi et al., 2020; Akiyama et al., 2024). The aim of current BRFD research is to develop a device that functions like a conventional FD in occluding and healing the aneurysm, but then completely resorbs into the body, eliminating or mitigating the risk of long-term complications.

Our group previously developed BRFDs out of magnesium and iron alloys. We found that the iron-based BRFDs outperformed their magnesium alloy counterparts in benchtop tests of flow diversion functionality and resorption rate. The iron-based BRFDs had a wire count, individual wire diameter, and overall device radial strength that was comparable to FDA approved FDs (Oliver et al., 2022b). Furthermore, we demonstrated MRI compatibility of the non-ferromagnetic iron-based BRFDs (Oliver et al., 2023). In the current work, we investigate the *in vivo* aneurysm occlusion efficacy of the iron based BRFD in the rabbit elastase induced aneurysm model. To our knowledge, this is the first time a braided iron-based BRFD has been evaluated *in vivo* for aneurysm occlusion efficacy.

## 2 Materials and methods

### 2.1 Devices

The BRFDs used in this study have been previously described in detail (Oliver et al., 2022b). In short, the BRFDs consisted of forty-eight braided wires, each with a diameter of 25 µm. Thirty-six of the wires were composed of a bioresorbable non-ferromagnetic iron-manganese-nitrogen (FeMnN) alloy (35% Mn, 0.15% N, balance Fe, by wt%) (Oliver et al., 2023; Schaffer, 2020). The remaining 12 wires were permanent and included to impart radiopacity. These wires were composed of tantalum coated in a thin layer of polyimide to prevent galvanic corrosion with adjacent FeMnN wires. The BRFDs were 4.75 mm in diameter and 7 mm in length.

### 2.2 Rabbit procedures

All animal experiments were approved by the Mayo Clinic Institutional Animal Care and Use Committee. Seven female New Zealand white rabbits weighing approximately 2.5–3 kg were used in this study. A sample size of seven rabbits was selected to match the sample size of previously reported investigations of FDA approved (Kallmes et al., 2009) and bioresorbable flow diverters (Akiyama et al., 2024; Sasaki et al., 2023) in the same rabbit model. Anesthesia was induced with an intramuscular injection of ketamine and xylazine. The rabbit was intubated, and anesthesia was maintained with 2%–3% isoflurane.

**FIGURE 1:**
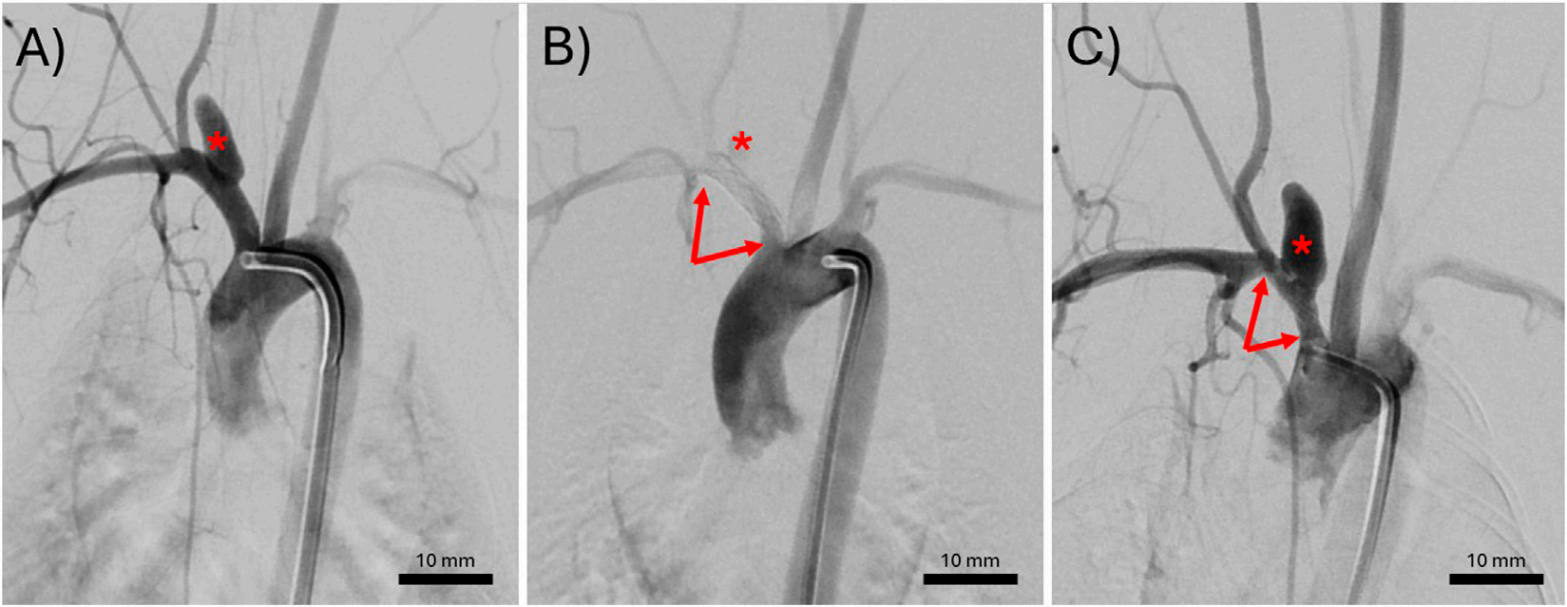
Representative digital subtraction angiograms of an elastase induced aneurysm **(A)** immediately before treatment, **(B)** immediately after treatment with a bioresorbable flow diverter (BRFD), or **(C)** at the 3-month follow up. The red asterisks indicate the location of the aneurysm. The red arrows point to the ends of a BRFD.

Elastase induced aneurysms were created in the rabbits using methods previously described in detail (Altes et al., 2000; Cortese et al., 2024). A surgical cutdown was used to gain vascular access with a 5F introducer sheath in the right common carotid artery (RCCA) approximately 3 cm cephalad from its origin. Under fluoroscopic guidance, a Fogarty balloon (Baxter Healthcare Corporation, Irvine, CA) was then advanced retrograde and expanded at the junction of the RCCA and the right subclavian artery to arrest flow into the RCCA. An aqueous porcine elastase suspension (100 Units/mL, Worthington Biochemical Corporation, Lakewood, NJ) was then injected into the RCCA in the dead space between the Fogarty balloon and the introducer sheath. The elastase solution was incubated for 20 min to degrade the arterial wall. The Fogarty balloon and sheath were then removed, and the RCCA was permanently ligated just proximal to the placement of the introducer sheath. The aneurysms were allowed to develop for a 3-week period, ultimately resulting in an aneurysm sac that was originally the base of the RCCA, with the right subclavian artery acting as the parent artery, as demonstrated in Figure 1A.

BRFDs were deployed at least 3 weeks after the aneurysm creation procedure. The rabbits received a daily dual antiplatelet therapy of 10 mg/kg of aspirin and clopidogrel, starting 2 days before and ending 30 days after the BRFD deployment procedure. A surgical cutdown was used to gain vascular access with a 5F introducer sheath in the right femoral artery. Under fluoroscopic guidance, a 0.035″ guidewire and guide catheter were navigated into the aortic arch and to the origin of the right subclavian artery. A digital subtraction angiography (DSA) was performed to assess the geometry and patency of the right subclavian artery and the elastase induced aneurysm. A BRFD loaded into a delivery catheter was then advanced into the right subclavian artery. The BRFD was deployed using a standard push-pull technique to deposit the BRFD in the right subclavian artery over the neck of the aneurysm. If necessary, balloon angioplasty was applied to ensure the BRFD was properly apposed to the vessel wall. A follow up DSA was taken to assess the placement of the BRFD.

Three months after BRFD deployment, the procedure described above was repeated to acquire a follow up DSA of the right subclavian artery, BRFD, and aneurysm. After the follow up DSA, the rabbit was euthanized with a lethal injection of pentobarbital. Immediately after sacrifice, the BRFD containing segment of the right subclavian artery and the aneurysm were excised and rinsed with heparinized saline to remove any postmortem clotting. The samples were stored in formalin for at least 24 h until histological analysis.

### 2.3 Angiographic outcomes

The neck, width, and height of the induced aneurysms were measured from the DSA taken immediately prior to BRFD deployment. The scale was adjusted using an external sizing marker with a known diameter placed in the field of view (Kallmes et al., 2009). The aneurysm dimensions are presented as the mean ± standard deviation. Aneurysm occlusion rate was assessed using the 3-month follow up DSAs. The degree of aneurysm occlusion was categorized as either completely patent, partially occluded, or completely occluded, as previously described (Kallmes et al., 2007).

### 2.4 Dissection microscope imaging

**FIGURE 2:**
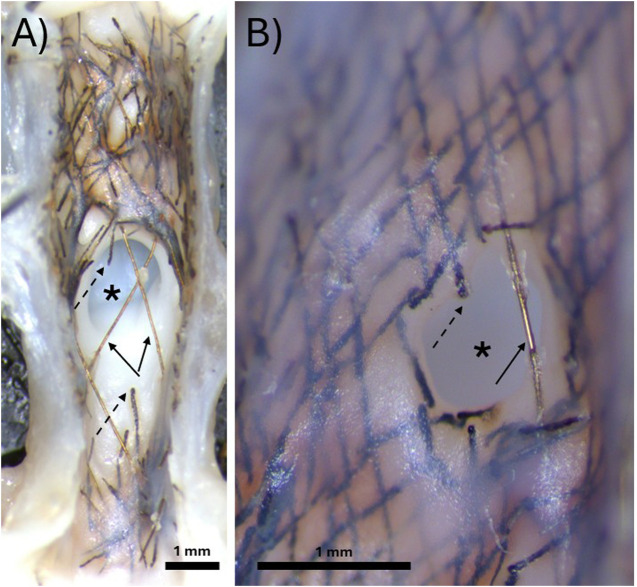
Representative gross dissection microscope images of bioresorbable flow diverters deployed over the aneurysm neck for 3 months. **(A)** Demonstration of a sample where no neointimal tissue developed over the aneurysm neck prior to the bioresorbable wires fragmenting away. **(B)** Demonstration of a sample where neointimal tissue developed over the bioresorbable wires and covered most of the aneurysm neck before the fragmentation of the remaining uncovered wires. In both images, the asterisk indicates the aneurysm neck, solid arrows indicate permanent radiopaque tantalum wires, and dashed arrows indicate bioresorbable FeMnN alloy wires.

**FIGURE 3:**
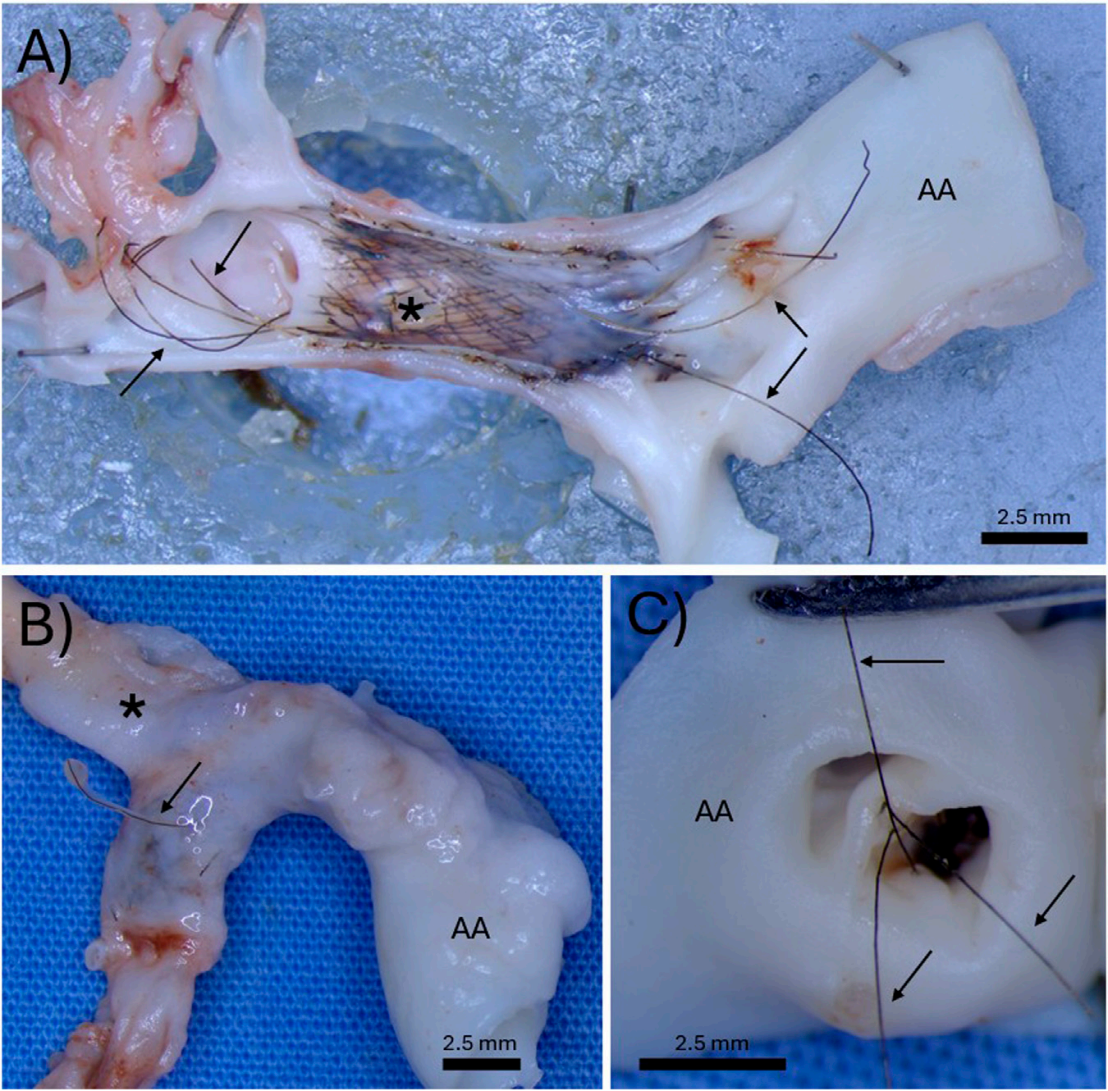
Three separate examples of the “harpoon” effect. **(A)** Demonstration of permanent tantalum wires springing out of the braid and extending in both directions past the original footprint of the bioresorbable flow diverter. **(B)** Demonstration of a permanent tantalum wire puncturing through the parent artery. **(C)** Demonstration of permanent tantalum wires extending and hanging into the aortic arch. The arrows indicate permanent tantalum wires, the asterisks indicate the location of the aneurysm, and the “AA” indicates the aortic arch.

Dissection microscope images of the samples were taken at 0.8–2.5X normal magnification (Leica MZ 125, Wetzlar, Germany). The bottom of the subclavian artery, opposite of the aneurysm, was cut longitudinally using surgical scissors. The sample was then spread open and pinned in place, as shown in Figures 2A, 3A. This allowed for imaging of the luminal surface of the BRFD and the aneurysm neck.

### 2.5 CD31 immunofluorescence

After dissection microscope imaging, the samples were washed three times in Tris-buffered saline (TBS) and incubated with 4% normal donkey serum for 1 h at room temperature. The samples were then incubated with CD31 primary antibodies (M0823, Dako, Carpinteria, CA) diluted 1:30 for an hour at room temperature and then overnight at 4°C. The next day, the samples were washed three times in TBS and incubated in secondary antibodies (Cy3-conjugated donkey anti-mouse, Jackson Immuno Research, West Grove, PA) diluted 1:200 for 2 h at room temperature. The samples were pinned open again, such that the luminal surface could be imaged with a confocal microscope (Olympus, Japan) at 10× or 20× normal magnification.

For validation, unstented regions of the parent artery served as positive CD31 controls. As a negative control, unstented regions of the parent artery were stained according to the protocol above, however the primary antibodies were replaced with just TBS. This allowed us to determine the degree of non-specific binding of the secondary antibodies, and ultimately confirm that the fluorescence signal was specific to CD31.

### 2.6 Hematoxylin and eosin

After confocal imaging, the samples were loaded into a tissue processor (Leica ASP 300S) where they were dehydrated in ethanol, cleared in xylene, and embedded in paraffin blocks. Then, using a technique previously described in detail (Dai et al., 2005), the sample-containing paraffin blocks were cut into Section 1 mm thick using a Isomet low speed saw (Buehler, Lake Bluff, IL). The metal wires were carefully removed from the tissue sections under the dissection microscope. The samples were then re-imbedded in a standard paraffin block and sectioned to 4 µm thickness.

The sections were deparaffinized and hydrated before staining with hematoxylin (Richard Allen Scientific, Kalamazoo, MI) for 5 min and Eosin for 1 min. The sections were then dehydrated in ethanol, cleared with xylene, and mounted onto microscope slides. The samples were imaged at 20× normal magnification using a slide scanner (Miotic Easy Scan Pro, Miotic Digital Pathology, San Francisco, CA).

## 3 Results

### 3.1 Angiographic outcomes

All the induced aneurysms were patent before BRFD deployment. The average aneurysm neck, width, and height were 3.06 ± 1.02 mm, 3.63 ± 0.67 mm, and 8.27 ± 1.87 mm, respectively. Balloon angioplasty was used during BRFD deployment in 3/7 rabbits. All the rabbits recovered after the BRFD deployment and survived until the 3 months follow up. Figure 1 demonstrates a representative DSA sequence of an aneurysm pre-treatment, immediately after BRFD deployment, and at the 3 months follow up. After 3 months, the aneurysms were completely patent in 5/7 rabbits, partially occluded in 1/7 rabbits, and in one rabbit, both the parent artery and the aneurysm had completely occluded in the follow up DSA. In the necropsy, it was determined that the subclavian artery had thrombosed.

### 3.2 Gross dissection microscopy

Figure 2 demonstrates two examples of gross dissection microscopy images of the aneurysm neck. In 3/7 rabbits, all the bioresorbable FeMnN wires over the aneurysm neck had fragmented away before they were covered with tissue and before they were able to occlude the aneurysm, as shown in Figure 2A. In 3/7 rabbits, some FeMnN wires were still present over the aneurysm neck. These wires had become covered with tissue, covering part of the aneurysm neck, as shown in Figure 2B.In both images, only the permanent tantalum wires remain over the unoccluded region of the aneurysm neck. The subclavian artery thrombosed in 1/7 rabbits, and therefore the aneurysm neck was not imaged. A thin neointima had developed over the wires of the BRFD adjacent to the parent vessel in both images.

Once the bioresorbable wires that make up most of the wires in the braid started to notably bioresorb, resulting in the loss of integrity of the tightly braided structure, the permanent tantalum wires sprung or “harpooned” out of the braid, elongating past the original length of the device. Figure 3A demonstrates an example of this harpoon effect, where the permanent tantalum wires have extended well past the original footprint of the BRFD on both ends. Figure 3B shows an example where the tantalum wires have sprung out of the braid and harpooned through the wall of the parent artery. Figure 3C shows an example where the tantalum wires have extended proximally past the device and ended up hanging in the aortic arch. Complications arising from the permanent Ta wires were observed in 5/7 rabbits. Of these 5 rabbits, 4 had wires extending past the original footprint of the device and 3 had Ta wires puncturing through the artery wall.

### 3.3 Histology

**FIGURE 4:**
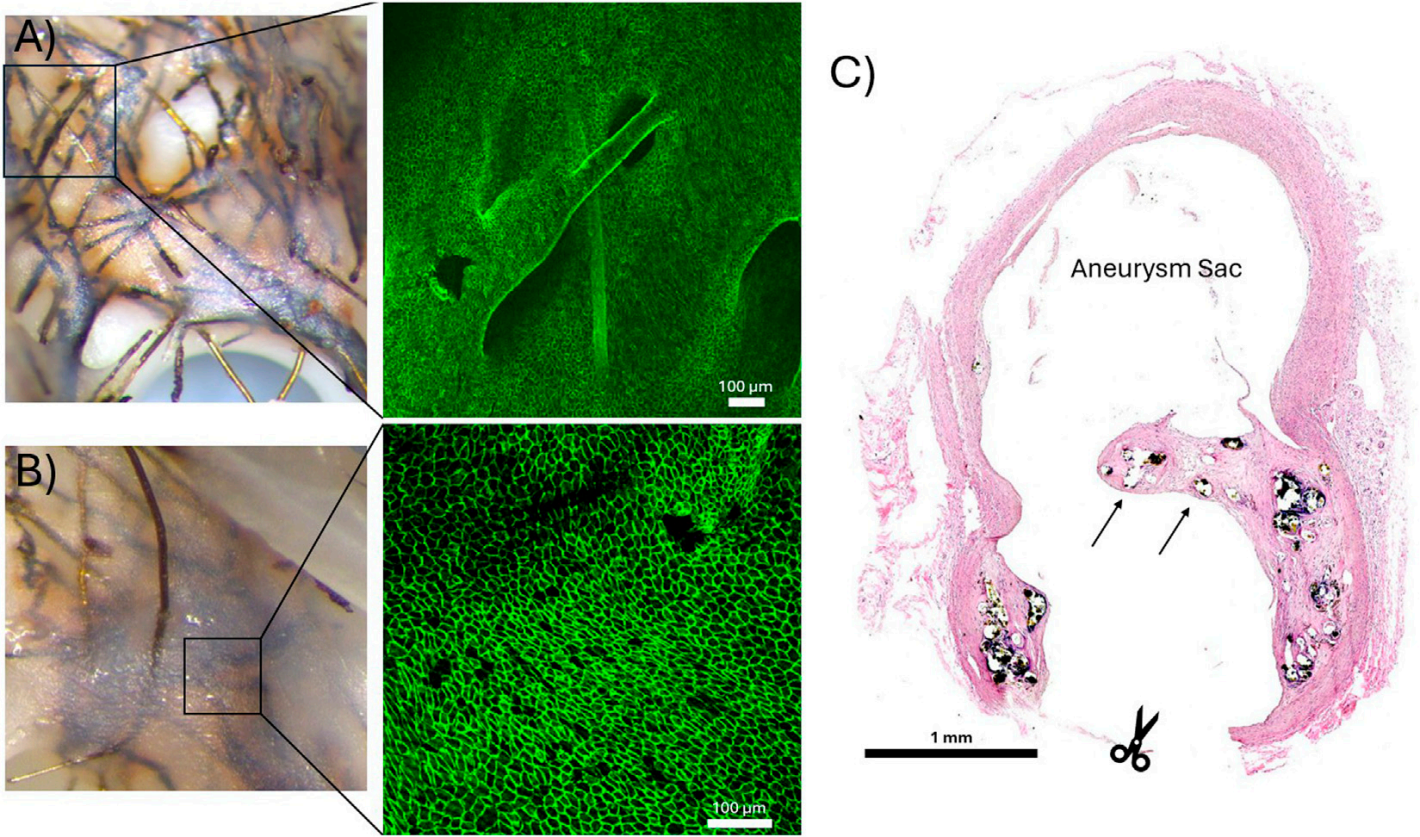
**(A, B)** *En face* confocal microscope images of a CD31^+^ endothelium developed over degrading FeMnN alloy wires. **(A)** Demonstration of CD31^+^ endothelial cells that have proliferated over an exposed FeMnN wire. The immunofluorescence image was taken at 10X normal magnification. **(B)** Demonstration of a confluent CD31^+^ endothelium lining the luminal surface of a neointima that has covered the bioresorbable flow diverter. The immunofluorescence image was taken at 20X normal magnification. **(C)** Representative H&E stained cross section of a bioresorbable flow diverter (BRFD) deployed over an aneurysm for 3 months. The arrows indicate where tissue has developed over BRFD wires across a large portion of the aneurysm neck. The scissor icon shows where the parent artery was cut during tissue processing to image the aneurysm neck with the dissection microscope.

A confluent CD31^+^ endothelium had developed over the bioresorbable FeMnN wires adjacent to the parent artery wall and across the regions of tissue that had developed over the aneurysm neck in the cases where FeMnN wires were still present. Figures 4A, B demonstrate examples of *en face* confocal microscopy images of the BRFDs stained with CD31 immunofluorescence. Figure 4A shows an example of a CD31^+^ endothelium growing over an FeMnN wire that is not fully encased within the neointima. Figure 4B demonstrates an example of a confluent CD31^+^ endothelium lining the luminal surface of a neointima that has formed over FeMnN wires. In both images, the CD31^+^ endothelial cells are in tight confluence and have a classic cobblestone morphology, indicative of a healthy matured endothelium. Figure 4C demonstrates a representative example of a hematoxylin and eosin-stained cross section of a BRFD deployed over the aneurysm neck, where FeMnN wires encased in tissue were present over the aneurysm neck after 3 months. In this example, tissue has developed over most of the BRFD wires over the aneurysm neck, covering most of the original neck.

## 4 Discussion

In this study, we found that BRFDs composed of the FeMnN alloy degrade too rapidly to effectively treat elastase induced rabbit aneurysms. The bioresorbable wires appeared to be fragmenting at the aneurysm neck and washing away prior to becoming encased in neointimal tissue and occluding the aneurysm. However, in all three cases where some FeMnN wires remained over part of the aneurysm neck after 3 months, the FeMnN wires were encapsulated in neointimal tissue. This suggests that the FeMnN wires, when present, were a suitable substrate for neointimal tissue development over the aneurysm neck. Taken together, we attribute the low aneurysm occlusion rate to material mechanical failure rather than an impaired biological healing response. Furthermore, we observed that the addition of some permanent radiopaque wires into a braided stent composed primarily of bioresorbable wires may result in dangerous outcomes. These findings are important for informing the development of improved BRFDs with a slower resorption rate and a different radiopacity approach.

The rabbit elastase induced aneurysm model has been used to evaluate FDs for over 15 years (Kallmes et al., 2007). A previous study in our lab investigated the efficacy of an early generation Pipeline Embolization Device (Medtronic, Dublin, Ireland) to treat elastase induced rabbit aneurysms of an almost identical geometry to the aneurysms in the current study (Kallmes et al., 2009). The study concluded that the Pipeline Embolization Device occluded 17/18 treated aneurysms with endpoints ranging from 1 to 6 months. This study serves as a benchmark for successful aneurysm occlusion efficacy in the rabbit elastase induced aneurysm model.

The FeMnN alloy used in this study degraded faster *in vivo* than anticipated. We previously evaluated the resorption rate of the same FeMnN BRFDs *in vitro* (Oliver et al., 2022b). The BRFDs were deployed in aneurysm models with straight parent vessels that were incorporated into a flow loop with Dulbecco’s Modified Eagle Medium flowing at a rate of 0.5 mL/s at 37°C. According to this analysis, we estimated that the FeMnN wires would completely dissolve after ∼9 months. Furthermore, we did not observe any fragmentation of FeMnN wires away from the aneurysm neck after 3 months of deployment in this model. A faster resorption rate *in vivo* than *in vitro* is opposite of what is typically observed in the bioresorbable metals literature. One potential reason for this discrepancy is a higher anticipated flow rate in the subclavian artery that the flow rate used in the *in vitro* model. Previous *in vivo* studies of bioresorbable pure and nitrided iron stents demonstrated that the device lifetime would be on the order 1–5 years (Peuster et al., 2006; Lin et al., 2017). However, the stent strut thickness in these previous studies ranged from 70 to 120 µm as opposed to the 25 µm diameter braided wires used in this study. Furthermore, the stents were deployed in straight arteries where all the struts were rapidly covered by neointima. In contrast, the BRFDs in this study were deployed in the curved right subclavian artery, with wires deposited over the aneurysm neck subjected to blood flow on all sides and delayed neointimal coverage. This may have contributed to the faster than anticipated resorption rate, particularly for wires deposited over the aneurysm neck. The impact on bioresorption rate of these two factors – 1) vessel curvature and resulting differences in shear stress exerted on different regions of the device from blood flow; and 2) strut position adjacent to the artery wall or over the aneurysm neck–needs to be studied in future experiments.

Several other groups are working to develop BRFDs out of polymers (Oliver et al., 2022a). Wang et al. developed a BRFD where approximately half the braided wires were bioresorbable poly glycolic acid and the remaining half were permanent nitinol (Wang et al., 2013). They reported a complete aneurysm occlusion rate of 75% when their device was evaluated in the rabbit elastase induced aneurysm model for 1.5–3 months. They found that their poly glycolic acid wires had completely resorbed by 3 months. The relatively high aneurysm occlusion rate with complete resorption of the poly glycolic acid wires was likely due to the high density of permanent nitinol wires remaining within the braid. Sasaki et al. developed a BRFD from braided bioresorbable poly (L-lactic acid) fibers, with permanent gold radiopaque markers placed at both device ends (Sasaki et al., 2023). Their BRFD maintained its braided structure out to 18 months and had a 48% complete aneurysm occlusion rate when evaluated in the rabbit elastase induced aneurysm model with endpoints ranging from 3 to 12 months. Morrish et al. developed BRFDs, called the ReSolv Flow-Diverting Stent, that were composed primarily of bioresorbable braided poly (L-lactic acid) wires with the addition of up to 8 permanent platinum-based wires within the braid to impart radiopacity (Morrish et al., 2024). They reported a complete aneurysm occlusion rate of 64.3% when evaluated in the rabbit elastase induced aneurysm model with endpoints ranging from 1 to 18 months. They did not report the *in vivo* resorption rate The polymeric BRFDs used in the studies above all featured individual wire diameters in the 40–50 µm range, which is larger than that of market approved flow diverters which range from 18 to 35 µm (Oliver et al., 2024; Oliver et al., 2022a; Jamshidi et al., 2020; Sasaki et al., 2023). Furthermore, the ReSolv Flow Diverting Stent’s reliance on several permanent metal wires for radiopacity and radial strength incorporated into the primarily bioresorbable braid may result in a similar harpoon effect observed in this study once the bioresorbable wires have notably degraded. This should be a focus of future long term *in vivo* studies. Akiyama et al. recently developed BRFDs composed of braided bare and poly-L-lactic coated magnesium alloy wires with individual wire diameter of 46 µm (Akiyama et al., 2024). These devices demonstrated suitable biocompatibility when evaluated in the rabbit abdominal aorta. However, their magnesium alloy wires had completely bioresorbed by 30 days. The ideal lifetime for a BRFD is still unclear. Clinically, ∼75% of aneurysms occlude after 6 months, ∼85% after a year, and ∼95% after 3–5 years (Becske et al., 2017). Consequently, we believe the ideal BRFD lifetime is ∼12 months to allow sufficient time for the aneurysm to heal while still imparting the advantages of bioresorbability. In the case of aneurysms that fail to occlude after several years, a BRFD may be advantageous by allowing for other retreatment options such as coiling or intrasaccular flow diverters.

One limitation of our study is that we did not include any permanent control FDs which could have served as a useful baseline for our aneurysm occlusion rate and histological assessments. However, many published manuscripts have evaluated the efficacy of permanent FDs in the rabbit elastase induced aneurysm model, including studies performed by our lab with market approved FDs (Kallmes et al., 2009). Another limitation of our methodology is that the catheter delivery systems used to deploy the BRFDs were made in house from repurposed clinical equipment. Consequently, not all the devices deployed with perfect wall apposition and 3/7 devices required balloon angioplasty. FD wall apposition is known to increase the rate of aneurysm occlusion (Rouchaud et al., 2016). Therefore, imperfect wall apposition in our study may have contributed to the low aneurysm occlusion rate. Future studies will utilize optimized custom delivery systems. Another limitation is that we did not perform a comprehensive analysis of our BRFD resorption rate, but rather just their ability to function in the application environment. Ongoing work includes evaluating the FeMnN BRFD resorption rate and uniformity when implanted in the rabbit aorta at serial endpoints. Future work will focus on investigating the role of vessel curvature and shear stress, as well as wire position (either adjacent to the vessel wall or over the aneurysm neck) on the resorption rate.

The results of this study emphasize the need for completely bioresorbable radiopaque materials to avoid requiring permanent radiopaque wires within the BRFD braid. The permanent presence of radiopaque wires may result in complications due to their migration after the bioresorbable components have degraded. To address this, our group recently developed drawn-filled-tubing (DFT) wires composed of an inner filament of radiopaque bioresorbable molybdenum surrounded by an outer layer of the FeMnN alloy (Griebel et al., 2024). Molybdenum has been shown to bioresorb at a very uniform rate *in vivo* (Sikora-Jasinska et al., 2021). Consequently, we believe next-generation BRFDs constructed from 25 µm diameter wires of this DFT material will have a slower, more uniform resorption rate, which may improve the fragmentation issue.

## 5 Conclusion

The FeMnN alloy used in this study to construct BRFDs degraded too quickly for the device to effectively treat rabbit elastase induced aneurysms. The FeMnN alloy wires degraded more rapidly than anticipated, which could have been caused by the vessel curvature and/or that the wires deposited over the aneurysm neck were exposed to blood flow on all sides. This work highlights the importance of developing materials for and evaluating them in models specific to device applications. Future work will focus on developing completely bioresorbable radiopaque materials with a slower resorption rate for the BRFD application.

## Data Availability

The original contributions presented in the study are included in the article/supplementary material, further inquiries can be directed to the corresponding author.
